# Diagnostic and prognostic role of TFF3, Romo-1, NF-кB and SFRP4 as biomarkers for endometrial and ovarian cancers: a prospective observational translational study

**DOI:** 10.1007/s00404-022-06563-8

**Published:** 2022-04-24

**Authors:** Hasan Turan, Salvatore Giovanni Vitale, Ilker Kahramanoglu, Luigi Della Corte, Pierluigi Giampaolino, Asli Azemi, Sinem Durmus, Veysel Sal, Nedim Tokgozoglu, Tugan Bese, Macit Arvas, Fuat Demirkiran, Remise Gelisgen, Sennur Ilvan, Hafize Uzun

**Affiliations:** 1 Department of Gynecologic Oncology, Health Science University, Cam Sakura Training and Research Hospital, Istanbul, Turkey; 2 Obstetrics and Gynecology Unit, Department of General Surgery and Medical Surgical Specialties, University of Catania, Via Santa Sofia 78, 95123 Catania, Italy; 3 Department of Gynecologic Oncology, Emsey Hospital, Istanbul, Turkey; 4 Department of Neuroscience, Reproductive Sciences and Dentistry, School of Medicine, University of Naples, Naples, Italy; 5 Department of Public Health, University of Naples Federico II, Via Sergio Pansini, Naples, Italy; 6 Department of Biochemistry, School of Medicine, Istanbul University-Cerrahpasa, Istanbul, Turkey; 7 Department of Obstetrics and Gynecology, Memorial Bahcelievler Hospital, Istanbul, Turkey; 8 Department of Gynecologic Oncology, Okmeydanı Training and Research Hospital, Istanbul, Turkey; 9 Department of Gynecologic Oncology, School of Medicine, Istanbul University-Cerrahpasa, Istanbul, Turkey; 10 Department of Gynecologic Oncology, American Hospital, Istanbul, Turkey; 11 Department of Pathology, School of Medicine, Istanbul University-Cerrahpasa, Istanbul, Turkey

**Keywords:** Biomarkers, Endometrial cancer, Ovarian cancer, TFF-3, Romo-1, NF-кB, SFRP4

## Abstract

**Purpose:**

This study aimed to evaluate trefoil factor 3 (TFF3), secreted frizzled-related protein 4 (sFRP4), reactive oxygen species modulator 1 (Romo1) and nuclear factor kappa B (NF-κB) as diagnostic and prognostic markers of endometrial cancer (EC) and ovarian cancer (OC).

**Methods:**

Thirty-one patients with EC and 30 patients with OC undergone surgical treatment were enrolled together with 30 healthy controls in a prospective study. Commercial ELISA kits determined serum TFF-3, Romo-1, NF-кB and sFRP-4 concentrations.

**Results:**

Serum TFF-3, Romo-1 and NF-кB levels were significantly higher in patients with EC and OC than those without cancer. Regarding EC, none of the serum biomarkers differs significantly between endometrial and non-endometrioid endometrial carcinomas. Mean serum TFF-3 and NF-кB levels were significantly higher in advanced stages. Increased serum levels of TFF-3 and NF-кB were found in those with a higher grade of the disease. Regarding OC, none of the serum biomarkers differed significantly among histological subtypes. Significantly increased serum levels of NF-кB were observed in patients with advanced-stage OC than those with stage I and II diseases. No difference in serum biomarker levels was found between those who had a recurrence and those who had not. The sensibility and specificity of these four biomarkers in discriminating EC and OC from the control group showed encouraging values, although no one reached 70%.

**Conclusions:**

TFF-3, Romo-1, NF-кB and SFRP4 could represent new diagnostic and prognostic markers for OC and EC. Further studies are needed to validate our results.

## Introduction

Ovarian cancer (OC) is the most lethal female reproductive tract malignancy worldwide, as reported by the National Cancer Institute (NCI), which estimates the death of 140,000 women every year [1]. Endometrial cancer (EC) is less lethal, since about 80% of cases are diagnosed in the early stages (I–II according to the International Federation of Gynecology and Obstetrics—FIGO) [2]. However, EC has the primacy of the most common gynaecological malignant disease, with an incidence rising alongside the growing prevalence of obesity. In 2018 over 380,000 new cases of EC and 295,000 new cases of OC have been registered with 89,000 and 184,000 deaths, respectively [3, 4].

Serum biomarkers can be used for screening, diagnosis, prognosis, or treatment monitoring of EC and OC, playing a fundamental role in primary and secondary prevention. Two different institutions have provided the definition of biomarkers: the NCI, which defines biomarkers as ‘a biological molecule found in blood, other bodily fluids, or tissues that is a sign of a normal or abnormal process, or of a condition or disease’ [5], and the World Health Organization (WHO), which defines them as ‘any substance, structure or process that can be measured in the body or its products and influence or predict the incidence of outcome or disease’ [6]. Recent discoveries of genetic pathways and tumor biomarkers in different types of cancers have opened new advances for using some of these biomarkers for the diagnosis, prognosis and as targets for emerging therapies. The oncosuppressor genes BRCA1 and 2 are the best-known genes involved in hereditary OC and breast cancer: indeed, they account for 70–80% of hereditary OC cases [7, 8]. Nowadays, numerous markers are already employed for the diagnosis and prognosis of OC. In addition, many others are being evaluated to predict progression as early as possible and thus find strategies to detect and prevent a recurrence [9].

CA125, human epididymis protein 4 (HE4), apolipoprotein A1, transthyretin, transferrin, and *β*2-macroglobulin are validated biomarkers used in the contest of Risk of Malignancy Index (RMI), Risk of Malignancy Algorithm (ROMA), OVA1 algorithms and International Ovarian Tumor Analysis (IOTA). These algorithms are used to distinguish benign diseases from malignant ones [10]. However, no biomarkers are currently used in daily medical practice for EC, though CA125 and HE4 can help prognosis and survival [11].

In this study, we evaluated the role of four proteins as biomarkers, still poorly investigated for OC and EC but which have aroused great interest in the context of other tumor diseases: trefoil factor 3 (TFF3), nuclear Factor kappa-light-chain-enhancer of activated B cells (NF-кB), secreted frizzled-related protein 4 (sFRP4), and Reactive Oxygen Species Modulator 1 (Romo-1).

TFF3 is an estrogen-regulated oncogene, member of the trefoil factor family that includes small proteins secreted and expressed by mucus secretory epithelia mainly in the gastrointestinal tract [12]. TFF-3 has been reported to be overexpressed both as a gene and as a protein in human neoplasms, including intestinal, pancreatic, and prostate cancers [13, 14] and be involved in gastric cancer progression [15]. Moreover, in breast cancer, the TFF-3 gene is regulated consistently by estrogen, and similar relations were also found in EC [16, 17].

NF-кB, a transcriptional factor involved in regulating the immune response and inflammation, is constitutively activated in several cancers, such as breast, colorectal, head and neck carcinomas. Growing evidence supports a significant role in oncogenesis due to the regulation of this transcriptional factor on the expression of genes involved in different processes, such as proliferation, migration, and apoptosis, related to cancer development and progression [18]. Furthermore, NF-kB may be responsible for reducing the efficacy of chemotherapy, inducing the expression of the multidrug resistance P-glycoprotein [18].

sFRP4 is part of a family of five secreted glycoproteins involved as modulators of Wnt signalling. Decreased expression or silencing of SFRP4 resulting in Wnt-pathway overactivation leading to inhibition of tumor cell apoptosis seems common in most, if not all, human cancers [19].

Romo-1 is upregulated in several cancers type. It is one of the most important proteins in the inner membrane of mitochondria involved in the production of Reactive Oxygen Species (ROS) by complex III of the mitochondrial electron transport chain. Romo-1 has been found in various neoplasm cells, responsible for the invasion and progression of cancer cells. Finally, Romo-1 seems to be associated with planned cell death (apoptotic pathways). Indeed, the increased expression of intracellular ROS promotes the release of cytochrome C of the mitochondria and triggers the caspases, resulting in cell death [20].

The purpose of our research was to investigate these molecules as diagnostic and prognostic biomarkers in preoperative samples of women with EC and OC to find their possible role in the management of these two cancers.

## Materials and methods

This prospective observational study was carried out at Cerrahpasa Faculty of Medicine, Division of Gynecologic Oncology, between April 2017 and May 2019. The study was approved by the Ethics Committee of Istanbul University-Cerrahpasa, School of Medicine (registration number: 83045809–604.01.02). In addition, written informed consent was obtained from all patients. The study was evaluated, selected and funded [funding number: TAB-2017–22506] by Istanbul University-Cerrahpasa, School of Medicine, Turkey, after rigorous peer-review.

The manuscript was prepared following the Strengthening the Reporting of Observational Studies in Epidemiology (STROBE) statement [21].

Preoperative serum samples were obtained from a nonconsecutive series of EC and OC patients. Exclusion criteria were defined as the presence of one or more of the following: (I) metastatic EC or OC; (II) patients who were operated in another clinic; (III) neoadjuvant chemotherapy or radiotherapy; (IV) secondary malignancy.

The same two gynaecological pathologists made all histopathological diagnoses: in case of disagreement, a further evaluation was required by a third pathologist. The histological type and stage of the disease followed FIGO classification [22, 23]. The charts and pathological findings were reviewed without knowing the preoperative TFF-3, Romo-1, NF-кB and SFRP4 values.

Among OC patients, maximal cytoreduction was defined as removing all gross tumor tissue with no visible disease. Optimal cytoreduction was defined as a residual volume of 1 cm or more minor after surgery. Residual tumor more than 1 cm was classified as suboptimal cytoreduction. Blood samples were collected in EDTA-containing tubes and anticoagulant-free tubes after an overnight fast. Plasma and serum were separated immediately and stored at -80ºC until analysis. After reaching the desired number of cases in both groups, all serum samples were melted at room temperature at the Medical Biochemistry Laboratory of Istanbul University-Cerrahpasa. Serum TFF3, Romo-1, NF-кB and SFRP4 concentrations were determined by commercial ELISA kits (Elabscience, USA), based on sandwich principle, according to the manufacturer’s instructions.

### Statistical analysis

Statistical analyses were performed using SPSS version 21. Patients’ characteristics and clinical features were summarized using standard descriptive statistics. Mann–Whitney *U* test was used for comparison between two groups. *T* test was used in the comparison of independent samples’ average. Receiver operating characteristics curves (ROC) were created for TFF3, Romo-1, NF-кB and SFRP4 serum concentrations as diagnostics for EC and OC by plotting sensitivity vs 1-specificity and area under the curve (AUC) was calculated for both markers. All *p* values were two-sided, and *p* < 0.05 were considered statistically significant.

## Results

A total of 101 patients were included in the study. The control group consisted of 30 women. There were 31 and 40 patients in EC and OC cancer groups, respectively.

Age, BMI and menopausal status were similar between control, EC and OC groups (Table 1).

**Table 1:** Comparison of clinical characteristics and level of serum markers between control and cancer groups

	Control group (*n* = 30)	Endometrial cancer (*n* = 31)	Ovarian cancer (*n* = 40)	*p*
Age, years	50.1 ± 12.3	54.1 ± 8.7	56.6 ± 11.9	NS
BMI, kg/m^2^	28.2 ± 4.1	31.0 ± 3.3	26.9 ± 3.6	NS
Postmenopausal	21 (70)	24 (77.4)	30 (75)	NS
TFF3, pg/mL	3.286 ± 0.253	3.672 ± 0.228	3.699 ± 0.194	0.01
Romo1, ng/mL	1.7 ± 0.2	2.6 ± 0.2	2.5 ± .0.2	0.01
NF-кB, ng/mL	2.1 ± 0.3	3.2 ± 0.5	3.2 ± 0.4	< 0.01
SFRP4, ng/mL	2.9 ± 0.5	2.2 ± 0.4	2.1 ± 0.3	0.05

Data are expressed as mean ± standard deviation or as frequencies (percentages)

*Abbreviations.* *BMI* Body Mass Index, *NF-кB* nuclear factor kappa B, *NS* not significant, *Romo1* reactive oxygen specific modulator 1, *SFRP4* secreted frizzled-related protein 4, *TFF3* trefoil factor 3

Serum TFF-3, Romo-1 and NF-кB levels were significantly higher in patients with EC/OC compared to control (TFF3: 3.699 ± 0.194 (mean ± SD) and 3.672 ± 0.228 in OC and EC (respectively) vs (vs) 3.286 ± 0.253 ng/mL in control group, *p* = 0.01; Romo1 2.5 ± 0.0.2 and 2.6 ± 0.2 in OC and EC (respectively) vs 1.7 ± 0.2 ng/mL in control group, *p* = 0.01; NF-кB 3.2 ± 0.4 and 3.2 ± 0.5 in OC and EC vs 2.1 ± 0.3 ng/mL in control group, *p* < 0.01).

Serum SFRP4 levels were significantly lower in cancer groups compared to the control group (2.1 ± 0.3 and 2.2 ± 0.4 in OC and EC (respectively) vs 2.9 ± 0.5 in the control group, *p* = 0.05). For more details, see Table 1.

**Fig. 1:**
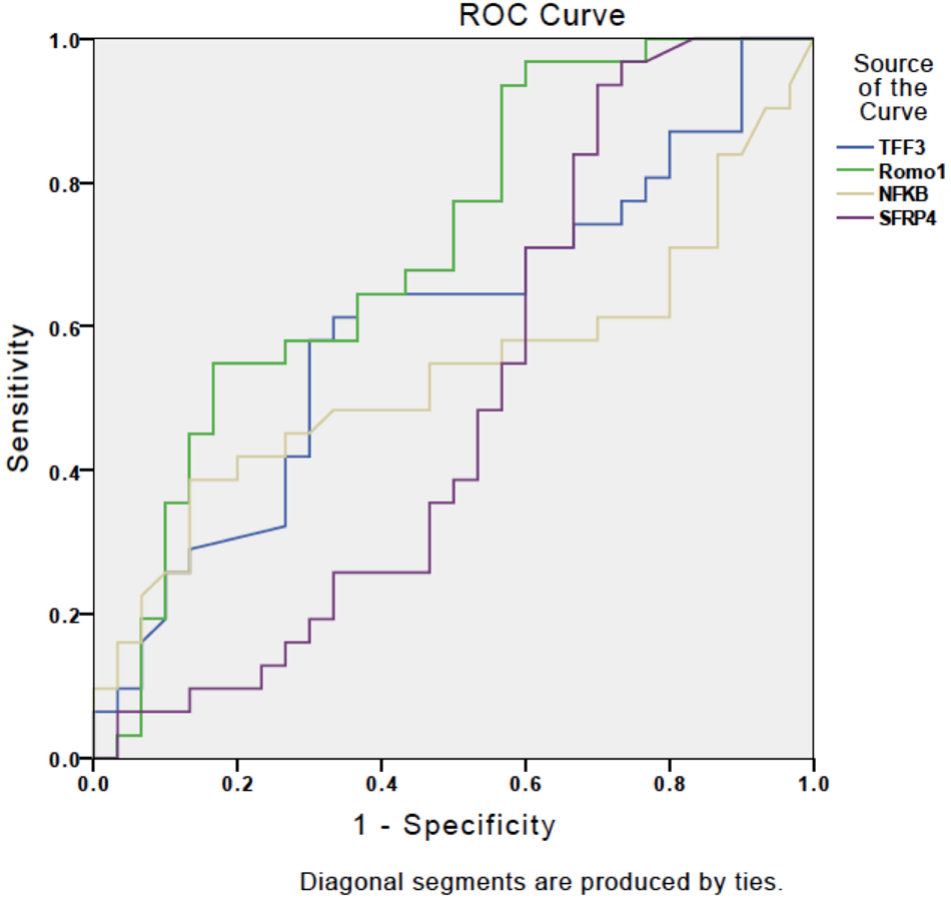
Receiver operating characteristic curves showing the performance of serum TFF3, Romo1, NF-кB and SFRP4 levels for differentiating between patients with and without endometrial cancer

As illustrated in Fig. 1, sensitivity and specificity of TFF3 for discriminating EC from the control group were 64.5% and 63.3%, respectively, when a cutoff serum TFF3 level of 3.559 ng/mL was applied. The same sensitivity and specificity values were found for Romo1 with a cutoff serum level of 1.9 ng/mL.

**Fig. 2:**
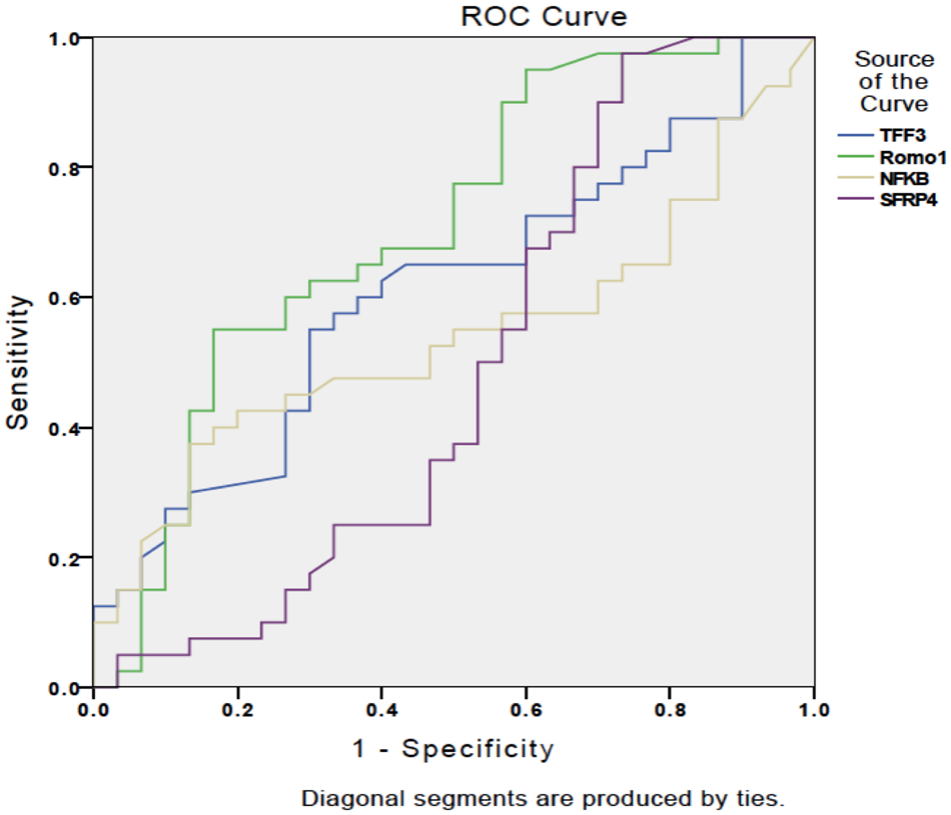
Receiver operating characteristic curves showing the performance of serum TFF3, Romo1, NF-кB and SFRP4 levels for differentiating between patients with and without ovarian cancer

**Fig. 3:**
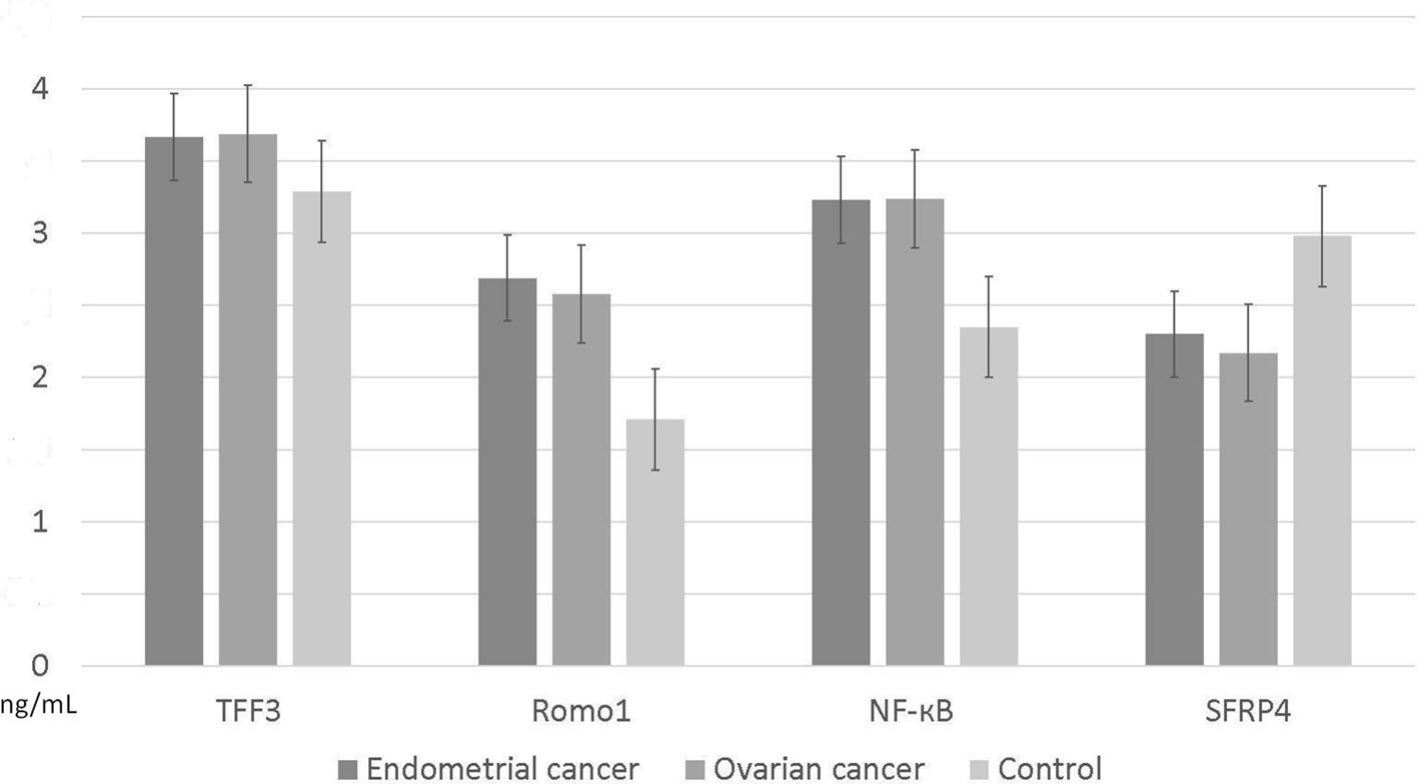
Histogram representing serum TFF3, Romo1, NF-кB and SFRP4 levels according to the EC, OC and control group. Data are expressed as mean ± standard deviation

As shown in Fig. 2, serum levels of Romo-1 and TFF3 were 1.8 and 2.682 ng/mL, respectively, while sensitivity and specificity for discriminating OC from the control group were, respectively, 67.5% and 60% for Romo-1, 62.5% and 60% for TFF3. All serum markers levels according to the EC, OC and control group are reported in Fig. 3.

All the other markers have sensitivity and specificity values below 60%. Therefore, the most sensitive and specific markers were Romo-1 and TFF3.

For more details about TFF3, Romo1, NF-кB and SFRP4 serum levels, sensitivity and specificity, see Figs. 1 and 2.

Patients with EC were separately analyzed (Table 2). Twenty-eight patients had an endometrioid EC, whereas three non-endometrioid (serous) EC. None of the serum biomarkers differed significantly between endometrioid and non-endometrioid EC. Eleven and 13 of 31 patients had stage IA and IB diseases, respectively. One patient had stage IIIA, and six patients had stage IIIC disease. Mean serum TFF-3 and NF-кB levels were significantly higher in advanced stages. In addition, increased serum levels of TFF3 and NF-кB were found in those with a higher grade of the disease. Only one patient had recurrence within a median follow-up time of 24 months (range 15–40).

**Table 2:** Clinical characteristics and serum TFF3, Romo1, NF-кB and SFRP4 levels of patients with endometrial cancer

	*n* (%)	TFF3, ng/mL	*p*	Romo1, ng/mL	*p*	NF-кB, ng/mL	*p*	SFRP4, ng/mL	*p*
Histology			NS		NS		NS		NS
Endometrioid	28 (90.3)	3.671 ± 0.211		2.6 ± 0.2		3.2 ± 0.4		2.3 ± 0.5	
Non-endometrioid	3 (9.7)	3.692 ± 0.42		2.6 ± 0.3		3.5 ± 0.7		2.0 ± 0.1	
FIGO stage			0.05		NS		0.02		NS
IA	11 (35.5)	3.381 ± 0.309		2.3 ± 0.2		2.5 ± 0.3		2.5 ± 0.2	
IB	13 (41.9)	3.619 ± 0.801		2.7 ± 0.3		2.9 ± 0.5		2.1 ± 0.3	
II	0	–		–		–		–	
IIIA	1 (3.2)	3.68		2.7		3.8		2.2	
IIB	0	–		–		–		–	
IIIC	6 (19.4)	4.418 ± 0.471		2.7 ± 0.2		3.9 ± 0.4		2.2 ± 0.4	
IV	0			–		–		–	
Histologic grade			0.01		NS		0.05		NS
1	8 (25.8)	3.32 ± 0.23		2.4 ± 0.3		2.5 ± 0.3		2.4 ± 0.3	
2	17 (54.8)	3.618 ± 0.35		2.7 ± 0.3		2.9 ± 0.5		2.3 ± 0.5	
3	6 (19.4)	4.333 ± 0.36		2.7 ± 0.3		3.9 ± 0.4		2.0 ± 0.1	
Recurrence			NS		NS		NS		NS
No	30 (96.8)	3.672 ± 0.245		2.6 ± 0.2		3.2 ± 0.4		2.2 ± 0.4	
Yes	1 (3.2)	4.301		2.7		3.1		1.7	

Data are expressed as mean ± standard deviation or as frequencies (percentages)

*Abbreviations*: *NF-кB* nuclear factor kappa B, *NS* not significant, *Romo1* reactive oxygen specific modulator 1, *SFRP4* secreted frizzled-related protein 4, *TFF3* trefoil factor 3

Table 3 shows the separate analysis of OC patients. The pathological subtypes of malignant ovarian tumors included serous cystadenocarcinoma (33), mucinous adenocarcinoma (3), endometrioid adenocarcinoma (3) and clear cell carcinoma (1). None of the serum biomarkers differed significantly among histological subtypes. Among OC patients, 85% had stage III and IV. Significantly increased serum levels of NF-кB were observed in patients with advanced-stage compared to those with stage I and II diseases. Three, 9 and 28 patients had grade (G) 1, 2 and 3 diseases, respectively. Higher serum levels of TFF3 and NF-кB were associated with the higher grade. Complete cytoreduction was achieved in 33 of 40 patients and was found to be correlated with lower NF-кB levels. The overall recurrence rate was 10% within a median follow-up time of 19 months (range 15–33 months). No difference in serum biomarker levels was found between those who had a recurrence and those who had not.

**Table 3:** Clinical characteristics and serum TFF3, Romo1, NF-кB and SFRP4 levels of patients with ovarian cancer

	*n* (%)	TFF3, ng/mL	*p*	Romo1, ng/mL	*p*	NF-кB, ng/mL	*p*	SFRP4, ng/mL	*p*
Histology			NS		NS		NS		NS
Serous	33 (82.5)	3.708 ± 0.17		2.6 ± 0.3		3.4 ± 0.4		2.0 ± 0.4	
Mucinous	3 (7.5)	3.662 ± 0.92		2.2 ± 0.2		2.5 ± 0.1		2.8 ± 0.3	
Endometrioid	3 (7.5)	3.671 ± 0.1		2.5 ± 0.2		3.1 ± 0.1		2.4 ± 0.4	
Clear cell	1 (2.5)	3.652		2.6		2.8		2.2	
FIGO Stage			NS		NS		0.05		NS
I	4 (10)	3.581 ± 0.197		2.2 ± 0.2		2.3 ± 0.1		2.6 ± 0.3	
II	2 (5)	3.672 ± 0.101		2.5 ± 0.1		2.4 ± 0.1		2.6 ± 0.3	
III	29 (72.5)	3.710 ± 0.14		2.6 ± 0.3		3.5 ± 0.4		2.1 ± 0.8	
IV	5 (12.5)	3.681		2.6 ± 0.2		3.8 ± 0.2		2.2 ± 0.1	
Histologic grade			0.05		NS		0.05		NS
1	3 (7.5)	3.593 ± 0.97		2.3 ± 0.1		2.4 ± 0.1		2.2 ± 0.3	
2	9 (22.5)	3.612 ± 0.139		2.4 ± 0.3		2.9 ± 0.3		2.1 ± 0.3	
3	28 (70)	3.711 ± 0.209		2.6 ± 0.5		3.5 ± 0.4		2.1 ± 0.4	
Residual tumor			NS		NS		0.05		NS
0	33 (82.5)	3.694 ± 0.192		2.3 ± 1.0		2.8 ± 0.4		2.1 ± 0.7	
≤ 1 cm	3 (7.5)	3.702 ± 0.120		2.3 ± 0.3		3.3 ± 0.2		2.1 ± 0.2	
> 1 cm	4 (10)	3.710 ± 0.913		2.5 ± 0.2		3.5 ± 0.4		2.1 ± 0.2	
Recurrence			NS		NS		NS		NS
No	36 (90)	3.695 ± 0.223		2.1 ± 0.3		3.0 ± 0.9		2.1 ± 0.4	
Yes	4 (10)	3.708 ± 0.101		2.1 ± 0.1		3.6 ± 0.3		2.1 ± 0.1	

Data are expressed as mean ± standard deviation or as frequencies (percentages)

*Abbreviations*: *NF-кB* nuclear factor kappa B, *NS* not significant, *Romo1* reactive oxygen specific modulator 1, *SFRP4* secreted frizzled-related protein 4, *TFF3* trefoil factor 3

## Discussion

To date, the use of molecular biomarkers in cancer research has made possible the identification of novel oncogenes/tumor suppressor genes that might be implicated in the development and progression of cancer, and that can be used as tumor biomarkers [24, 25]. Indeed, the panorama of oncological therapies in recent years has been revolutionized by the molecular study of cancer, which has allowed new therapeutic strategies, the so-called targeted therapy, and immunotherapy. In this respect, Poly (ADP-ribose) polymerase (PARP) inhibitors (PARPi), initially used only for OC patients with mutations in BRCA1/2, are the most representative example of targeted therapy [26]. In the BRCA mutation carriers, the role of CA125 evaluation has been described in two different settings by Grandi et al.: in the first one, the need for an integrated clinical work-up including CA125 dosage, ultrasound and computed tomography (CT) examination for the early detection of OC [7]; in the second one, in BRCA 1/2 mutation carriers undergoing risk-reducing salpingo-oophorectomy, where CA125 level reduction has been found only partially associated with surgery [8]. However, few markers are currently available and used in endometrial and ovarian cancer [27, 28]. Over the years different risk factors have been associated with the development of EC rather than OC, such as hormonal therapy, smoke, obesity, the latter always associated with EC: a recent study has confirmed how an increase in BMI is associated with endometrial rather than ovarian cancer, but both serous and endometrioid histotypes [29]. Although CA125 is commonly used in the clinic for these reasons, it is endowed with low sensitivity and specificity [30, 31]. Nevertheless, it still represents a useful serum marker to early differentiate between OCs and BOTs with higher sensitivity for stage I endometrioid OC compared to other OC histotypes [32]. Thus, in the last years, several researchers tried to define new molecules that can be helpful in the diagnosis and or prognosis of these tumors. Identifying new prognostic factors besides new therapeutic approaches may help distinguish different biological subgroups. This is particularly important for patients who develop recurrent disease in gynaecological tumors. Future efforts should focus on establishing more targeted and individualized treatment strategies in biologically distinct subgroups.

Many authors have employed array-based genome-wide discovery platforms to identify aberrant mRNA expression and somatically acquired DNA sequence variants or mutations to determine the molecular changes underlying the development of OC and EC as a first step to identify molecular markers with potential clinical utility [33, 34]. Using this technology, some proteins such as TFF3, Romo-1, NF-кB and SFRP4 have been identified as potential for diagnostic and prognostic targets in EC and OC [18, 35–38].

According to this evidence, in our study, we collected serum TFF3, Romo-1, NF-кB and SFRP4 concentrations to determine their levels in patients affected by OC and EC. We discovered that serum TFF3, Romo-1 and NF-кB levels were significantly higher in patients with EC or OC compared to those without cancer instead of serum SFRP4 levels that were significantly lower in cancer groups compared to the control one. We also noticed that mean serum TFF3 and NF-кB levels were significantly higher in advanced stages, while none of the serum biomarkers differed significantly between those who had a recurrence and those who had not. These results seem to be in accordance with literature data. Studies suggest that the TFF3 may play a role in different functions, such as proliferation, migration and angiogenesis, processes that when altered are crucial for tumorigenesis [37, 39]. Indeed, TFF3 has been reported to be overexpressed at the gene and the protein level in human neoplasms and associated with poor prognosis. TFF3 alterations have been demonstrated also in gynecological cancers, such as endometrial and ovarian tumors [17, 40]. In light of this, TFF3 may play a role in regulating cancer progression by increasing tumor metastasis by promoting anti-apoptosis, pro-invasive and angiogenesis agents [41]. In a study by Bignotti et al., a significantly higher serum TFF3 level in endometrioid EC patients when compared with healthy women or patients with endometrial hyperplasia was found [36]. Moreover, serum TFF3 levels showed higher sensitivity in the detection of patients with G3-endometrioid EC when compared with CA125 levels. This evidence may support the design of prospective studies evaluating the potential of TFF3 as a new tool for pre- and post-operative surveillance of EC patients. In our study, increased serum levels of TFF3 were found in patients with a higher grade of the disease both in EC and OC.

In a more recent study, Neubert et al. analyzed TFF3 levels in 89 who women underwent hysteroscopy and endometrial biopsy for postmenopausal bleeding [42–45], showing how TFF3 levels were significantly higher in patients with endometrial carcinoma compared with endometrial hyperplasia group [46]. Pandey et al., in a study conducted to evaluate the role of tamoxifen in the EC, observed that elevated TFF3 protein expression was found in EC but not in normal endometrial tissue, and its elevated expression in EC cells increased oncogenicity, invasiveness and tumor growth, as well as myometrial invasion. Moreover, it explained how tamoxifen's implication in overexpression of TFF3 in EC cells was critical in promoting EC progression [47]. As regards these data, prospects can be focused on evaluating of inhibition of TFF3 function to limit the progression of EC. El-Balat et al. analyzed the expression of TFF3 in a cohort of 137 borderline tumors of the ovary (BOT): none of the serous and endometrioid BOT showed strong TFF3 expression. On the contrary, a higher TFF3 expression was found in BOT mucinous histology and BOT with mixed histology, suggesting a potential function of the protein in these histological subtypes [37]. Expression analysis of TFF3 was performed in a cohort of 91 OC patients by Hoellen et al. No significant difference in TFF3 expression was found based on age, FIGO stage or residual tumor; meanwhile, a significant correlation of TFF3 expression and grade was detected [48]. However, since few studies have been carried out on TFF3 expression and its role in EC and OC and, mostly, few studies have considered its prognostic value, TFF3 expression deserves further investigation.

The idea of analyzing the expression of Romo1 in EC and OC comes from the evidence of its role in the process of invasion and also the progression of cancer cells [35, 48]. It is involved in normal cellular processes, such as cell proliferation, senescence, and death. As ROS regulating protein, Romo1 is associated with the level of oxidative stress and the production of ROS in cancerous cells, considering that one of the most important causes for the incidence of cancer is the increase of free radicals and ROS [49]. Moreover, oxidative stress-induced Romo-1 expression is associated with tumor cell invasion via NF-κB signaling has been reported to increase constitutive activation of NF-κB in hepatocellular carcinoma [50]. Therefore, oxidative stress, promote tumor cell invasion through Romo-1 expression and constitutive NF-κB activation. For these reasons, it is reasonable to assume Romo-1 as a promising therapeutic target for diseases characterized by NF‑κB deregulation. The evidence in our study of Romo-1 overexpression in OC and EC makes this molecule susceptible to more detailed and wider research.

Further evidence about the role of NF-κB, as a key link between inflammation and cancer, and its increased activity has been reported in several types of cancer. The activation of NF-κB signaling can occur through canonical or non-canonical pathways which have distinct roles in tumor progression; moreover, cancer cells have been shown to produce different proinflammatory and proangiogenic substances in direct response to NF-κB activity, as found in OC [51]. Indeed, in OC, the deregulated NF-κB activity promotes chemoresistance, cancer stem cell maintenance, metastasis and immune evasion. Although NF-κB is an attractive target in OC, current therapeutic strategies are limited due to unwanted side effects, caused by wide inhibition of this major signaling pathway in normal physiological and immunological cellular functions. For these reasons, next research may be enforced to suppress NF-κB only in the tumor cell population of OC and concurrently activate canonical NF-κB signaling in immune cells to promote and support anti-tumor immunity.

Finally, the finding of altered expression in serum level of SFRP4 evaluated in our study, resulting in a lower serum SFRP4 expression in cancer groups compared to the control one, seems to agree with recent literature data [52]. SFRP4 is a putative modulator of the Wnt signaling pathway, important in cell proliferation, and may be implicated as a tumor suppressor: indeed, under normal conditions, SFRP4 can function as a suppressor of cell growth and variations in the expression level of SFRP4 has been found in many tumors, such as endometrial, cervical, ovarian, prostate, bladder, colorectal, mesothelioma, pancreatic, renal, and oesophageal tumors [53, 54]. In a study conducted by Pohl et al., it has been found that SFRP4 expression is decreased in the normal endometrium when compared to EC cells and from the analysis made in serous OC cells, SFRP4 protein expression is decreased, predicting a poorer outcome and prognosis [38, 53, 55].

## Strengths and limitations

A limitation of our study is represented by the small number of patients analyzed and the heterogeneity of the population sample (regarding age, tumor histotype and stage of the disease).

Despite the aforementioned limitations, there are several strengths of our study: the presence of a control group and its prospective design.

## Conclusions

Nowadays, non-invasive methods for diagnosis and prognosis of EC and OC are needed and there is growing interest in the evaluation of the role of specific serum biomarkers. Up to date, many studies try to define are the best molecules to analyze, preferring those with high sensitivity and specificity. The CA125 assay remains a useful, low-cost and easy tool for the initial and follow-up evaluation of OC patients as well as the assessment of BRCA mutation carriers in patients with high risk or initial diagnosis of OC. This paper tries to play a role in this scenario, evaluating serum TFF3, Romo-1, NF-кB and SFRP4 concentrations to determine their levels in the EC and OC patients: in our case, the patients with EC and OC had TFF3, Romo-1 and NF-кB serum levels significantly higher and SFRP4 serum levels lower compared to the control one. Their sensibility and specificity for discriminating EC and OC compared to the control group show encouraging values, although no one reaches 70%. Future prospective and randomized trials are needed to define new biomarkers which could also help to identify specific markers for molecular targets therapy [26].
